# Validity of the patient health questionnaire 9-item in autistic youths: a pilot study

**DOI:** 10.1186/s12888-021-03556-w

**Published:** 2021-11-12

**Authors:** Thanita Pilunthanakul, Tze Jui Goh, Daniel Shuen Sheng Fung, Rehena Sultana, John Carson Allen, Min Sung

**Affiliations:** 1 Ministry of Health, 16 College Rd, College of Medicine Building, Singapore, 169854 Singapore; 2 Department of Child & Adolescent Psychiatry, Institute of Mental Health, Buangkok Green Medical Park, 10 Buangkok View, Singapore, 539747 Singapore; 3 Duke-NUS Medical School, l8 College Rd, Singapore, 169857 Singapore

**Keywords:** Depression adolescent PHQ-9 autism ROC

## Abstract

**Background:**

Autistic adolescents have greater predisposition to depression and suicidality than neurotypical adolescents. Early detection is essential for timely treatment. The Patient Health Questionnaire 9-item (PHQ-9) is a brief screen for depression. The study examines the validity of the PHQ-9 for detecting major depressive disorder (MDD) in autistic youths.

**Methods:**

English speaking youths aged 10–18 years, with DSM-IV/DSM-5/ICD-10 diagnosis of Autism Spectrum Disorder (ASD), and their parents presenting to a child psychiatric service were invited to participate between May 2018 to August 2020. Participants completed the respective self- and parent-rated PHQ-9 independently. MDD was verified using the MINI-Kid (Mini-International Neuropsychiatric Interview, Kid version).

**Results:**

One hundred one youth, mean (SD) age 14.6 (2.3), were enrolled. 27 (27%) met criteria for current MDD. Mean total PHQ-9 scores, percentage ratings for severity of symptoms of depression, functional impairment, dysthymia and suicidality were compared. Areas under the ROC curve and statistically optimal cutoffs were determined. Parents rated depressive symptoms severity lower than their children. The PHQ-9 displayed low sensitivity with high false negative rates at conventional, adjusted and proposed cutoffs.

**Conclusions:**

Future studies should improve on the validity and reliability of existing depression screening tools, or develop more appropriate screening methods of depression, for autistic youths.

## Background

Depression is a common comorbid psychopathology in autism spectrum disorders (ASD) [1, 2]. A prospective study over a period of 1–5 years investigating psychiatric comorbidities in autistic youths aged 6 to 20 years reported that 42% had experienced an increase in expression of suicidal behaviors from childhood to adolescence, and of the 30% that suffered from major depressive disorder (MDD), 20% exhibited severe suicidal behavior or had attempted suicide [3].

Screening for depression is essential for early detection and treatment. Families of autistic patients are more likely to make frequent primary care visits [4], thus, primary care physicians play an important role in diagnosis, and prompting early intervention for depression—especially for patients and families who may not seek psychiatric care. Current tools used to assess depression in autistic youths may be inappropriate for outpatient healthcare settings as they may be costly, time-consuming, involve extensive scoring processes and/or an additional psychiatric specialist assessment [5–8].

The Patient Health Questionnaire 9-item (PHQ-9) is a widely used depression screen [9, 10] in primary care, outpatient, and research settings with good reliability and validity. It is brief, user-friendly, easy to administer, and available in parent- and self-reported formats. Furthermore, the PHQ-9 promptly categorizes the severity level of depressive symptoms and is available without cost, making it effective for busy healthcare settings. The PHQ-9 has shown good validity in neuro-typical adolescents [11], in a multi-ethnic Asian sample such as those in Singapore [12] and in autistic adults [13]. However, no study has investigated its utility and optimal cutoff in autistic youths as yet.

This study aims to determine the optimal cutoffs for the self- and parent-reported PHQ-9 screen for MDD (as verified by the Mini-International Neuropsychiatric Interview, Kid version [MINI-Kid]) in autistic youths. We hypothesize that different cutoffs (compared to neuro-typical adolescents) may be needed to exclude MDD in autistic youths, while maintaining an acceptable false positive/negative rate, since clinical symptoms of ASD may overlap with core symptoms of depression (e.g., sleep disturbances, abnormal eating habits, psychomotor retardation and irritability) [14]. Secondary aims include determining internal consistency and convergent validity between the self- and parent-rated PHQ-9 and investigating possible correlations between participant characteristics and severity of functional impairment and depressive symptoms in autistic youths.

## Method

### Participants

Patients who attended consultations at the outpatient Child Guidance Clinic (CGC), Institute of Mental Health (IMH), Singapore, from May 2018 to August 2020 were invited to participate in the study if they fulfilled the following inclusion criteria: (i) aged 10 to 18 years, (ii) diagnosed with ASD based on the Diagnostic and Statistical Manual, 5th (DSM-5) or 4th Edition (DSMIV), or International Statistical Classification of Diseases and Related Health Problems, 10th Revision (ICD-10), by a registered psychiatrist, (iii) a Clinical Evaluation of Language Fundamentals, 5th Edition, Screening Tool (CELF-5) [15], age equivalent score above 8 years of age to ensure comprehension of questions on the PHQ-9, and (iv) parental consent and agreement to participate. The exclusion criteria were as follows: (i) patients who were unable to speak, non-responsive, non-communicative or had not acquired language, ii) patients with co-occurring Intellectual Disability, and (iii) caregivers who were unable to read and understand English.

The a priori sample size was predicated on the assumption of 15% MDD prevalence [1]. Our intention was to recruit 100 ASD patients, of which 15 were expected to have MDD, in order to achieve ≥80% power at α = 0.05 to detect target sensitivity of 86%.

### Materials

The PHQ-9 [16] is a multipurpose instrument used in screening, diagnosing and monitoring the severity of depression. The optimal cutoff of 11 in neuro-typical adolescents typically indicates clinically significant depressive symptoms with 89.5% sensitivity and 77.5% specificity [11]. Respondents rate the frequency of the following nine symptoms over the previous two-week period on a four-point Likert scale (never = 0, several days = 1, more than half the days = 2, nearly every day = 3): depressed mood, anhedonia, sleep problems, feelings of tiredness, changes in appetite or weight, feelings of worthlessness, difficulty concentrating, feelings of sluggishness or worry and suicidal ideation. Scores of 5–9, 10–14, 15–19, and 20–27 represent mild, moderate, moderately severe, and severe depression, respectively. The PHQ-9 also has screening questions for dysthymia and suicidality, as well as a functional impairment item that examines the degree of interference with daily functioning due to the indicated symptoms.

The MINI-Kid [17] is a structured diagnostic interview for DSM-5 disorders in children and adolescents. It can be administered in a shorter time with similar validity and reliability compared to extended clinical interviews like the Kiddie Schedule for Affective Disorders and Schizophrenia and has high interrater reliability. Studies have used the MINI-Kid to assess for comorbid psychiatric disorders in children and adolescents with ASD [18, 19]. Patients fulfilling at least five of the DSM-5 criteria during the previous two weeks (with at least one symptom as depressed mood or loss of interest) were determined to have met criteria of MDD. Other comorbid psychiatric disorders were also assessed as part of the interview.

The demographic form included participant age, sex, ethnicity, education level, medications, relationship to child, and parental job status.

### Procedure

Participants and parents who met the inclusion criteria were enrolled. After providing written informed consent, youth completed the CELF-5 conducted by a trained research team member to determine eligibility. Enrolled participants completed the demographic form and the self- and parent-rated PHQ-9 (English versions) independently. Subsequently, a research team member, trained by a qualified clinical psychologist and blinded to the PHQ-9 results, administered the MINI-Kid (English version) to both youth and parent. Recruitment, consent, and administration of all questionnaires and interviews were completed on the same day. No additional visits were required. The study protocol was approved by the Institutional Research Review Committee (CRC Ref 612–2018) and the National Health Group Domain Specific Review Board (DSRB Ref 2018/00352).

Receiver operating characteristic (ROC) analysis was performed to determine sensitivity, specificity, positive (PPV) and negative predictive value (NPV), and likelihood ratios for various cutoffs of the self- and parent-rated PHQ-9. The Youden Index (YI; J-statistic, calculated as sensitivity + specificity − 1) was also employed to identify a statistically ‘optimal’ cutoff. The reliability of the PHQ-9 versions was evaluated using Cronbach’s α. Convergent validity and associations were assessed using Pearson (*r*) and Spearman (*r*_*s*_) correlation coefficients for continuous and ordinal variables, respectively. McNemar’s test was used to compare the difference between self- and parent-reported severity of depressive symptoms, functional impairment, dysthymia, and suicidality. Statistical significance was set at *p* < 0.05. Descriptive statistics and analysis were conducted using SPSS Version 24.0.

## Results

The final sample consisted of 101 youth and parent pairs. Youth participants ranged in age from 9 to 18 years old, with mean (SD) age of 14.6 (2.3); CELF-5 scores ranged from 11.0 to 30.0, with mean (SD) 21. (5.2). Participant characteristics are summarized in Table 1.

**Table 1:** Demographic and clinical characteristics of study sample. Data contributed by author

Characteristics of adolescent participants (*n* = 101)	n (%)
Sex	
Male	88 (87.1)
Female	13 (12.9)
Ethnicity	
Chinese	85 (84.2)
Malay	6 (5.9)
Indian	4 (4.0)
Others	6 (5.9)
Education Level	
Primary	11 (10.9)
Secondary	66 (65.4)
Special Education Secondary	13 (12.9)
Tertiary	11 (10.9)
On psychotropic Medications	46 (45.5)
Number of comorbidities (based on MINI-Kid)	
0	33 (32.7)
1	35 (34.7)
2	18 (17.8)
> 2	15 (14.9)
Characteristics of parent participants (*n* = 84)	n (%)
Relationship to child	
Mother	84 (83.2)
Father	17 (16.8)
Ethnicity	
Chinese	85 (84.2)
Malay	7 (6.9)
Indian	4 (4.0)
Other	5 (5.0)
Parent's highest education level	
Below secondary	4 (4.0)
Secondary	38 (37.6)
Tertiary	59 (58.4)
Parent’s job status	
Unemployed	39 (38.6)
Part-time	2 (2.0)
Full-time	60 (59.4)

MINI-Kid, Mini International Neuropsychiatric Interview for Children and Adolescents

**Fig. 1:**
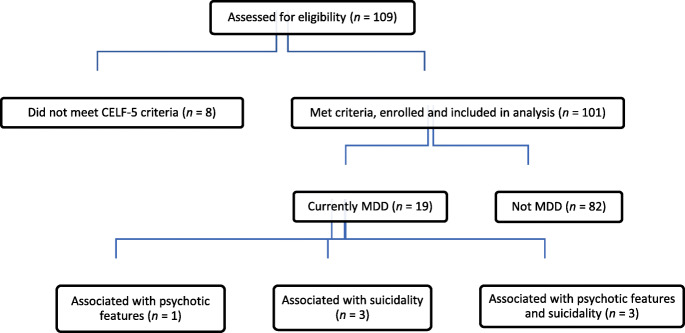
Flow of participants through study. CELF-5, Clinical Evaluation of Language Fundamental, 5th Edition, Screening Tool; MDD, major depressive disorder

Of the 101 youths in the final sample, a total of 27 (27%) had experienced a MDD or MDE (major depressive episode; in cases diagnosed with Bipolar Disorder), at least once in their lifetime. 19 (19%) met the criteria for MDD, as verified by the MINI-Kid. Of these 19 cases, 12 (63%) experienced recurrent MDEs, and/or associated suicidality and psychotic features (Fig. 1). Those currently diagnosed with MDD were subsequently flagged up to their primary psychiatrist for management.

**Fig. 2:**
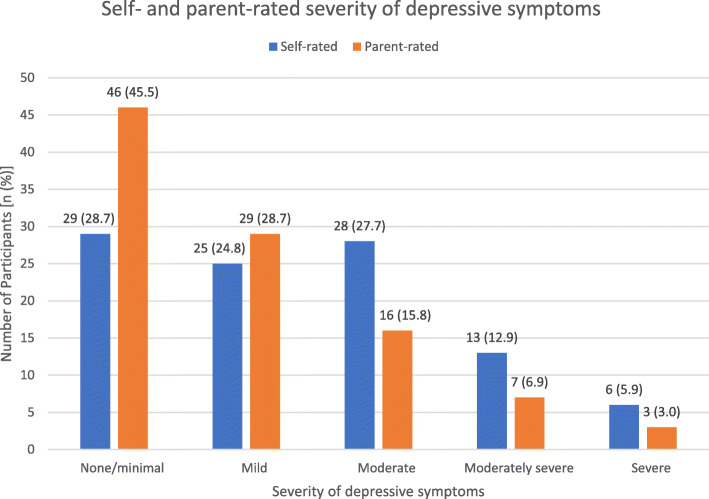
Self- and parent-rated severity of depressive symptoms (*p* = 0.0071*, n = 101*). Data contributed by author

**Fig. 3:**
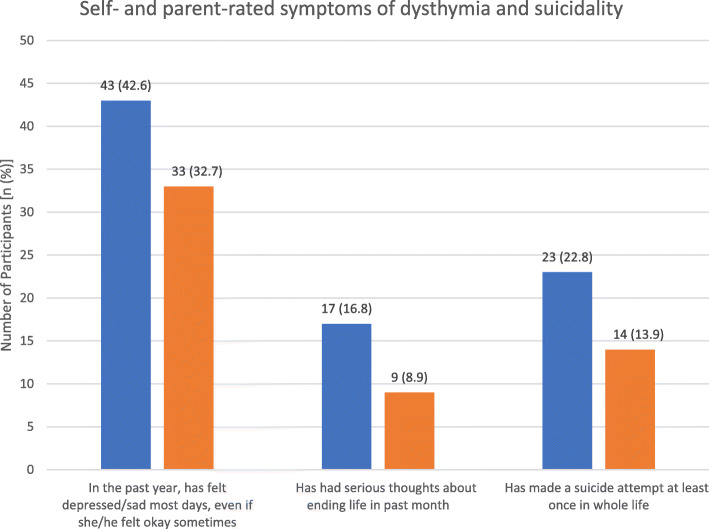
Self- and parent-rated symptoms of dysthymia during the past year (*p* = 0.1228), suicidal thoughts in the previous month (*p* = 0.0522) and attempted suicide at least once in lifetime (*p* = 0.0495). *n* = 101. Data contributed by author

**Fig. 4:**
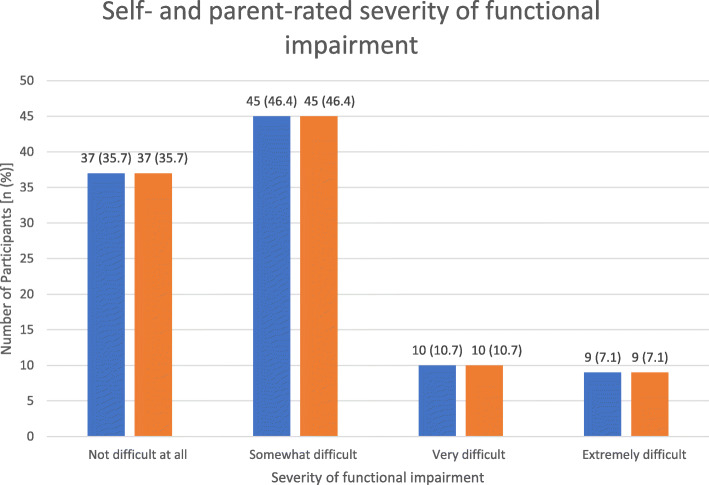
Self- and parent-rated severity of functional impairment (*p* = 0.3949*,* n *= 101)*. Data contributed by author

The mean (SD) total scores for self- and parent-rated PHQ-9 were 9.0 (6.0) and 6.2 (5.6) respectively. Frequencies of depressive symptoms severity were compared between self- and parent reports (*p* = 0.0071), summarized in Fig. 2. Frequencies of dysthymia during the past year (*p* = 0.1228), suicidal thoughts in the previous month (*p* = 0.0522) and attempted suicide at least once (lifetime; *p* = 0.0495) were also compared and summarized in Fig. 3. Frequencies of functional impairment severity levels were compared and consistent between self and parent reports (*p* = 0.3949), summarized in Fig. 4.

**Fig. 5:**
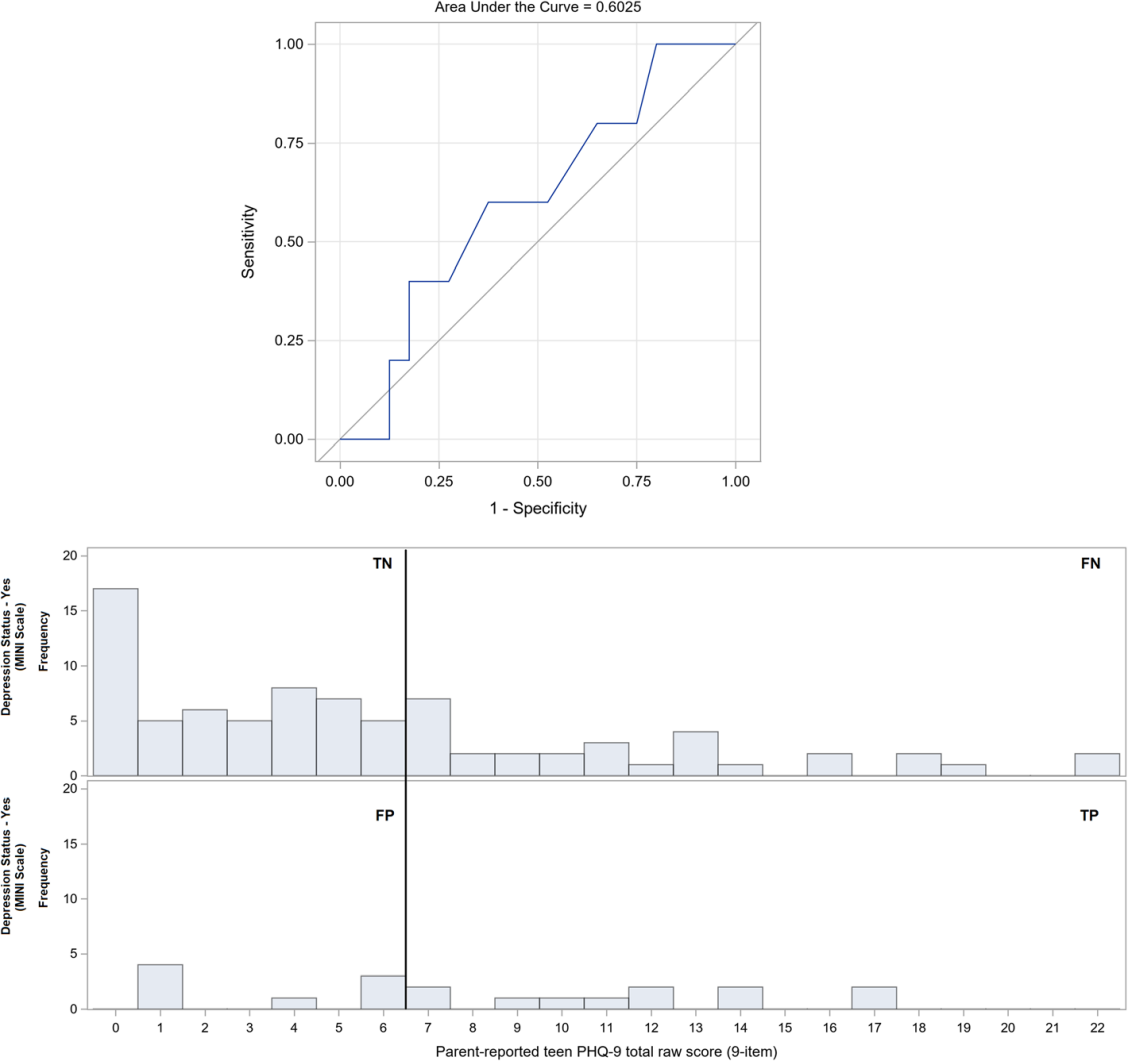
ROC curve (above) of parent-rated PHQ-9 for detecting MDD (AUC: 0.653, 95% CI: 0.521–0.784, *p* = 0.094) and corresponding histogram (below). PHQ-9, nine-item teen Patient Health Questionnaire; ROC, receiver operating characteristic; TN, true negative; FN, false negative; FP, false positive; TP, true positive. Data contributed by author

**Fig. 6:**
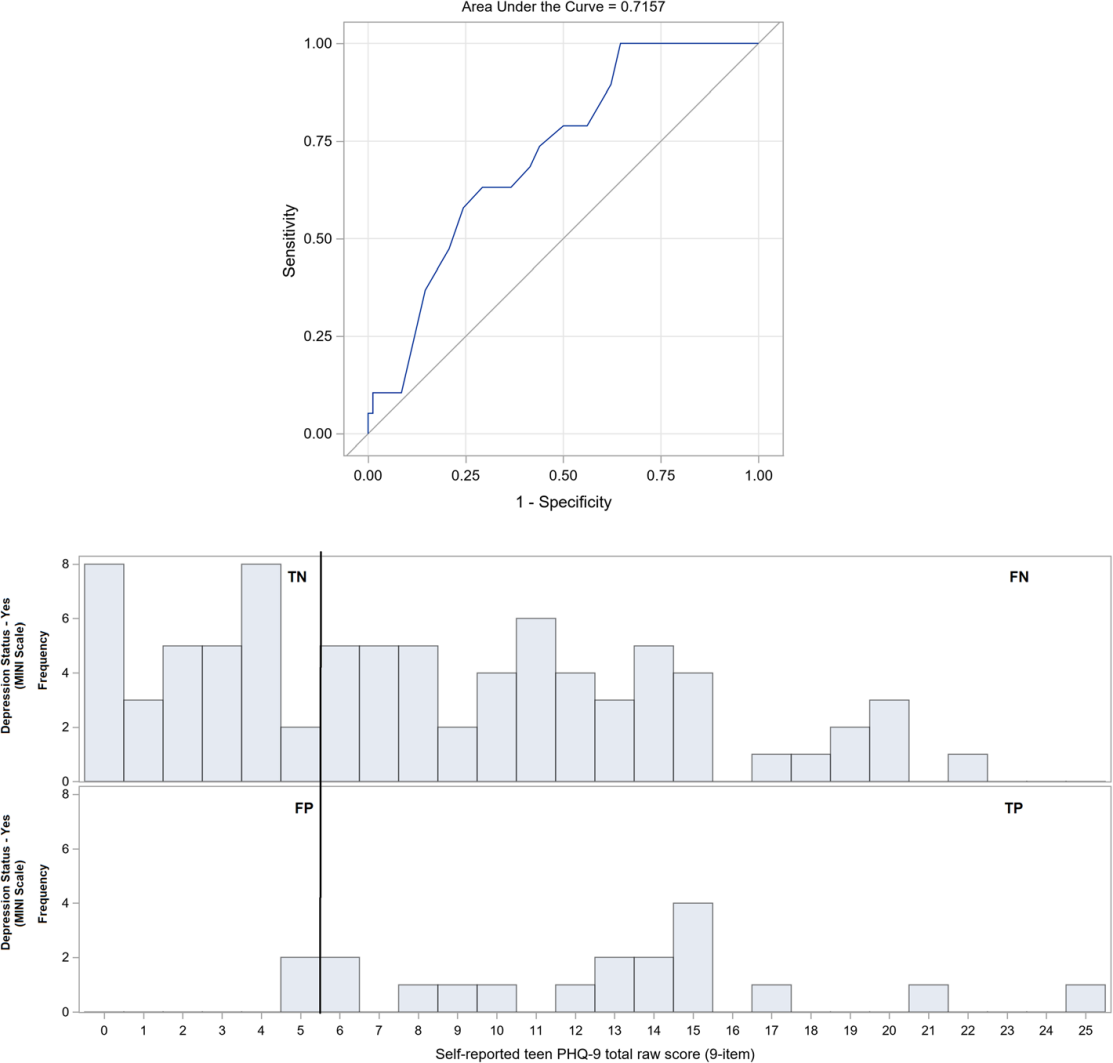
ROC curve of self-rated PHQ-9 for detecting MDD (AUC = 0.716, 95% CI: 0.600–0.831, *p* = 0.007) (above) and corresponding histogram (below). PHQ-9, nine-item teen Patient Health Questionnaire; ROC, receiver operating characteristic; TN, true negative; FN, false negative; FP, false positive; TP, true positive. Data contributed by author

Table 2 lists selected cutoffs based on the ROC analyses of self- and parent-rated PHQ-9 scores for diagnosing MDD in autistic youths as verified by the MINI-Kid. ROC curves are presented in Figs. 5 and 6. The area (95% CI) under the curve (AUC) for the parent- and self-rated PHQ-9 was 0.653 (0.52–0.78, *p* = 0.094) and 0.716 (0.60–0.83, *p* = 0.007), respectively. The statistically optimal cutoffs based on the YI was 5 for the self-rated PHQ-9 with 100% sensitivity and 35% specificity, and 6 for the parent-rated PHQ-9 with 74% sensitivity and 59% specificity. For the self-rated PHQ-9 with YI cutoff of 5, the negative predictive value (NPV) was 100%, and the positive predictive value (PPV) was 26%; for the parent-rated PHQ-9 with YI cutoff of 6, the NPV was 91%, and the PPV was 29%.

**Table 2:** Sensitivity, specificity, and positive and negative predictive values for a diagnosis MDD across various cutoffs of the PHQ-9 (*n* = 101)

Cutoff Score	Sensitivity (%)	Specificity (%)	Positive Predictive Value (%)	Negative Predictive Value (%)	Youden’s Index
Self-rated					
3	100.0	19.5	22.4	100.0	0.195
4	100.0	25.6	23.8	100.0	0.256
5	100.0	35.4	26.4	100.0	0.354
6	89.5	37.8	25.0	93.9	0.273
7	78.9	43.9	24.6	90.0	0.229
8	78.9	50.0	26.8	91.1	0.289
9	73.7	56.1	28.0	90.2	0.298
10	68.4	58.5	27.7	88.9	0.270
11	63.2	63.4	28.6	88.1	0.266
12	63.2	70.7	33.3	89.2	0.339
13	57.9	75.6	35.5	88.6	0.335
14	47.4	79.3	34.6	86.7	0.266
15	36.8	85.4	36.8	85.4	0.222
Parent-rated					
3	78.9	34.1	21.7	87.5	0.131
4	78.9	40.2	23.4	89.2	0.192
5	73.7	50.0	25.5	89.1	0.237
6	73.7	58.5	29.2	90.6	0.322
7	57.9	64.6	27.5	86.9	0.225
8	47.4	73.2	29.0	85.7	0.205
9	47.4	75.6	31.0	86.1	0.230
10	42.1	78.0	30.8	85.3	0.202
11	36.8	80.5	30.4	84.6	0.173
12	31.6	84.1	31.6	84.1	0.157

Grey: the proposed cutoff. Orange: the statistically optimal cutoff based on the Youden’s Index. Data contributed by author

Both self- and parent-rated PHQ-9 showed acceptable internal consistency, as demonstrated by the Cronbach α coefficients of 0.810 and 0.845, respectively. Although statistically significant, the Pearson correlation between self- and parent-rated PHQ-9 scores would be considered weak to moderate (*r* = 0.491, *n* = 101, *p* = 0.01).

Spearman correlation coefficients for associations between self- and parent-rated severity of depressive symptoms and of functional impairment, and number of comorbidities are summarized in Table 3. Age and education demonstrated no significant correlation with self- or parent-rated severity of functional impairment or with depressive symptoms**.**

**Table 3:** Spearman correlation coefficients for associations between self- and parent-rated severity of depressive symptoms and of functional impairment, and number of comorbidities (*n* = 101)

		Self-reported severity		Parent-reported severity		No. of comorbidities
		Functional impairment	Depressive symptoms	Functional impairment	Depressive symptoms	
Self-rated severity	Functional impairment		0.534**	0.287**	0.252*	0.338**
	Depressive symptoms	0.534**		0.428**	0.410**	0.327**
Parent-rated severity	Functional impairment	0.287**	0.428**		0.583**	0.285**
	Depressive symptoms	0.252*	0.410**	0.583**		0.307**
No. of comorbidities		0.338**	0.327**	0.285**	0.307**	

**Correlation significant at *p* = 0.01 level (2-tailed)

*Correlation significant at *p* = 0.05 level (2-tailed)

## Discussion

Consistent with previous literature [6, 20, 21], our study demonstrated a high co-occurrence of depression in autistic youth, emphasizing the importance of routine screening for depression. Almost half of our total sample self-reported dysthymia symptoms and at least a quarter reported suicidality. 19% met criteria for MDD, and of this sample, 37% had associated psychotic symptoms and/or suicidality (Fig. 1). The high rate of depression may be attributed to ASD-related social issues, such as peer victimization [22], social difficulties [23, 24], impaired ability to emotionally adapt to perceived social failures [25], greater loneliness [26], poorer quality of life [27] and lack of friendships [28] compared to neuro-typical peers. Additionally, autistic individuals may engage in greater use of maladaptive forms of emotional regulation, such as rumination and shutting down (e.g. emotional numbing) [29], which are highly associated with the development of depression and anxiety.

Similar to previous literature [7, 8], the number of comorbidities was found to be positively correlated to self-reported functional impairment (*r*_*s*_ = 0.338; *p* = 0.01) and depressive symptoms (*r*_*s*_ = 0.327; *p* = 0.01); this suggests that effective treatment of comorbid psychiatric issues may be important for a holistic approach to depression in autistic youth.

Although the PHQ-9 has been shown to have similar psychometric properties within both the autism and community sample aged 15–80 years old, indicating its usefulness in measuring depression in ASD individuals in that age group [13], our study found it to be a less than ideal screening tool for MDD in autistic youths. High sensitivity is of primary concern in determining the clinical utility of depression screens when false negative outcomes can mean morbidity and fatality. Using the conventional cutoff of 11 for the self- and parent-rated PHQ-9 in autistic youth in this study yielded lower sensitivities and specificities than for neuro-typical adolescents [11] (Table 2). The statistically optimal cutoffs based on the YI for the self- and parent-rated PHQ-9 are 5 and 6, respectively. However, despite these adjustments, the parent- and self-rated PHQ-9 would still be inappropriate depression screens. A sensitivity of 73.7% for a cutoff of 6 in the parent-rated PHQ-9 would lead to a high false positive (58.5% specificity) and negative rate. Even if sensitivity was increased to 78.9% by decreasing the cutoff to 4, the specificity decreases even lower to 40.2%. On the other hand, a self-rated PHQ-9 cutoff of 5 yields 100% sensitivity, but an even higher false positive rate (26.4% specificity). A proposed cutoff of 8 would only minimally increase specificity to 50.0% while compromising sensitivity to 78.9%. Nonetheless our results show relatively poor specificity compared to sensitivity at the adjusted cutoffs for both self- and parent-rated PHQ-9 in detecting MDD in autistic youths, indicating that the PHQ-9 would be relatively better at ‘ruling out’ MDD than ‘ruling in’. Hence, a positive screen on the PHQ-9 could be followed up with a formal supplementary evaluation by a specialist familiar with the manifestation of depression in autistic youths.

Other studies also found relatively poor specificity compared to sensitivity in detecting co-occurring emotional problems in autistic youths at established optimal cutoffs [8]. These findings may be due to depressive symptoms overlapping with baseline characteristics of ASD itself [14, 30], making it difficult to distinguish between the two. Another possible reason for the lack of specificity is the high co-occurrence of other psychiatric comorbidities in ASD, which could account for some symptoms in the PHQ-9 [31].

Possible reasons for lower PHQ-9 cutoffs for autistic youth compared to neuro-typical adolescents are: 1) emotional states not being congruent to what is observed, 2) PHQ-9 not fully capturing the manifestation of depression in autism, 3) alexithymia in ASD [32] and/or 4) difficulty interpreting questions asked [33]. Previous literature suggest that individuals with ASD find identifying, processing [34], regulating [29], and communicating [35] emotions challenging. They also often have marked impairment in the quality of the affect—showing facial expressions that are neutral and difficult to interpret—and disengagement that could mute the association with depressive symptoms [29]. Inadequate emotional regulation abilities could predispose them to rapid escalation of reactions that are out of proportion to what is typically experienced. Taken together with poorer and unconventional communication skills, these could have resulted in depression, despite a lower PHQ-9 score. Another possibility for a lower score is that ASD-specific symptoms, such as changes in repetitive and stereotypic behaviors, are not accounted for. Hence, it may be difficult for current tools to fully capture depression experienced by ASD youth. A screen that incorporates an evaluation of change in clinical symptoms of ASD during the onset of depression, such as intensification of social withdrawal and ritualistic behavior/obsessions coupled with associated features, such as irritability, compulsivity, hyperactivity and decreased adaptive functioning, [30, 36] may be a helpful and appropriate alternative.

Possible reasons for the discrepancy between the parent- and self-rated adjusted cutoffs could be related to parents’ under-detection of depressive symptoms in autistic youth and cultural stigma around mental illness [12, 37]. Our findings indicate almost twice as many youth rating their depressive symptoms as moderate, moderately severe, and severe compared to their parents; this under-reporting by parents is consistent with previous studies [38]. Parents may be less forthcoming when reporting depressive symptoms, less inclined to discuss depression with their child, or simply may not be aware of the indications of depression. Parents may also interpret these symptoms as temporary variations in typical autistic behavior. In this regard, if the autistic youth self-reports depressive symptoms that are significant while their parent does not, it may be prudent to evaluate the case further regardless.

Our study result is preliminary, and our sample size may be too small to support definitive conclusions. Replication of our analyses and evaluation of psychometric properties of the PHQ-9 on a larger and more diverse sample with variables that could be related to depression in autistic youth can improve our understanding of the appropriate uses and interpretations of the PHQ-9 in specific subgroups within the autistic population. For example, males with ASD are found to have more externalizing behavior problems [39] and less internalizing symptoms [40] than females, potentially leading to positively skewed results. Further studies should also explore how depression may present in those with lower communication and cognitive abilities, as these factors could have variably affected and influenced our results. Additionally, 46% of our patients were on psychotropic medications at the time of enrollment, which could affect the presentation of depression. Those on antidepressants for more extended periods may have less florid depressive symptoms and functional impairment, while those on other psychotropic medications, such as anxiolytics or stimulants, may have had medically-induced subthreshold depressive symptoms. There is also a possibility of recall bias when interviewing autistic participants about MDEs and symptoms of other psychiatric comorbidities. For example, since this population may have a greater tendency to ruminate over past negative experiences [41], reported depressive symptoms/recollections might extend beyond the previous two weeks indicated for diagnosis. Controlling for the impact of confounding factors such as psychotropic medications, sex, ethnicity, age, comorbidities and history of previous MDEs in future studies [42] is recommended.

Future studies could also examine the influence of clinical severity profile of participants’ ASD and its consequential functional impairments, on the presentation of co-occurring depression. Severe impairment in social skills may lead to increased dysfunction in interpersonal relationships and negative life events, precipitating relatively more depressive symptoms. On the other hand, severe ASD symptoms could mask the manifestation of depression symptoms, resulting in under-reporting in both youths and parents, or a missed diagnosis by the clinician. A depression screening scale that takes into account the severity of ASD symptoms and corresponding changes would be useful to determine the presentation, and severity of presentation of depression in individuals with ASD.

## Conclusion

Due to the high co-occurrence of depression in autistic youth, routine screening, exploration of functional impairment, and effective treatment of other comorbid psychiatric issues should be conducted to allow for early multidisciplinary intervention, and minimization of associated morbidity and suicide risk. Although our study found low sensitivities with unacceptably high false negative rates using conventional, statistically optimal, and proposed cutoffs for the parent- and self-rated PHQ-9, the small sample size does not allow definitive cutoffs to be made with confidence. The discrepancy between proposed parent- and self-rated cutoffs indicate that interpretations based on a single source may be inconclusive and require additional evaluation by a specialist. Fundamentally, it is difficult to evaluate depression in the autistic population as 1) patients may have difficulty in communicating and understanding how they feel, and 2) behaviors suggesting a depressive disorder in ASD have yet to be clearly identified [14, 30]. Future studies should improve on the validity and reliability of existing screening tools, or explore and develop more appropriate screening methods for depression in autistic youths.

## Data Availability

The datasets used and/or analyzed during the current study are available from the corresponding author on reasonable request.

## Abbreviations

ASD: Autism Spectrum Disorder
AUC: area under the curve
CELF-5: Clinical Evaluation of Language Fundamentals, 5th Edition, Screening Tool
CGC: Child Guidance Clinic
CRC Ref: Clinical Research Committee Reference
DSM-5: Diagnostic and Statistical Manual, 5th Edition
DSMIV: Diagnostic and Statistical Manual, 4th Edition
DSRB Ref: Domain Specific Review Board Reference
FN: false negative
FP: false positive
TN: true negative
TP: true positive
IMH: Institute of Mental Health
MINI-Kid: Mini-International Neuropsychiatric Interview, Kid version
MDD: Major Depressive Disorder
MDE: Major Depressive Episode
NPV: negative predictive value
PHQ-9: Patient Health Questionnaire 9-item
PPV: positive predictive value
ROC: Receiver Operating Characteristic
YI: Youden Index
